# East and West

**Published:** 1887-11-12

**Authors:** Leslie Keith

**Affiliations:** Author of "The Chilcotes," "Uncle Bob's Niece," etc.


### East and West.

By Leslie Keith.

Author of "The Chilcotes," "Uncle Bob's Niece," etc.

(*Continued from p.* 105.)

Chapter VII.—An Explorer.

Yet Kitty could not shake off the memory of her visit, even within the next few days; it haunted her and spoiled the closing triumphs of the season. She wished fervently that she had not gone, and that was perhaps the reason why she proposed to go again.

"It would be rather fun to go down there and stop for a week or so," she said, addressing the assembled family at dessert, when the servants were out of hearing. Jack being as good as one of the family, was present.

"'Pon my word," said that young man, "it's a great idea. Newer than a house-boat, or a canal voyage or a caravan."

"One might take lodgings," said Kitty, emboldened by this approval, "and observe the manners and customs of the natives, and then write a book about them. That is what everybody does who goes to a new country. No, Jack; I didn't say I wanted you."

"I will take lodgings opposite you, and also notes."

"I should want to furnish my rooms. They don't seen to have got the length of furniture down there. Papa, you will pay my travelling expenses?"

Those charming books "All Sorts and Conditions of Men," and "Children of Gibeon" had not been written, so that Kitty can not be accused of plagiarism when she evolved this idea from her inner consciousness. In a pause of her chatter, Alicia delivered judgment.

"It is an excellent idea," she said, graciously. "When papa and I go away"—Miss Frankland had already decided that her papa's health required an immediate visit to Homburg—"you cannot do better than carry it out; but you must take someone older and more responsible than a maid with you."

"I'll look after her," said Jack boldly.

Kitty looked, as indeed she felt, exceedingly disconcerted. It is one thing to announce an audacious scheme when you are perfectly certain that it cannot be carried out, and another to be taken at your word. Kitty would have liked the comfort of feeling that her generous impulses were only checked by family opposition, but by what strange miracle had Alicia come to range herself on her sister's side? Kitty could well have spared this offered alliance, for she was not of the stuff out of which explorers are made.

"Papa hasn't consented," she said feebly.

"He wouldn't hinder you in so good a work," said the elder daughter confidently.

Mr. Frankland looked from one to the other of them with his quiet smile. What an excellent father this was to do just what he was told to do!

Thereafter Alicia took a most flattering interest in the scheme, and tendered much sound advice to the traveller. "You had better get some gowns," she said, "something useful, and dark and ugly. You won't want pretty things, as I shall, because I don't suppose Bethnal Green is like Homburg. I think I know of an old woman who will do to look after you. She is quite a duenna. Jack, of course, is out of the question, and you mustn't think of taking Robins. She is far too young and pretty. Besides, you won't want the services of a maid down there."

Thus Kitty felt herself daily more and more committed to her scheme and daily more given over to melancholy. She saw no hope of escape. Papa only laughed, and pointed out that Alicia must be obeyed, and Jack, detestable boy! actually declared it would be very good fun. Up to the last day before the travellers were to set out for Homburg she was allowed to suffer the full retribution of her rashness, and then relief came most unexpectedly from the quarter whence no mercy had seemed possible. Alicia walked into her sister's room at bedtime. Kitty was ruefully contemplating a garment whose severity held out scant promise of becomingness.

"Jack will hate me in it," she said dolefully.

"Then you had better tell Bonner to give it away. You will not require it at Homburg."

"It is you who are going to Homburg," said Kitty with vehemence. "Why do you torment me?"

"No, you are going instead of me. You never really wished to go down to the East-end, though you were quite willing we should all think you very heroic for proposing it," said this severe moralist. "I shall go there, since one of us must go."

"But I've no clothes," Kitty pouted; "you made me get those horrid things."

"I've no doubt you can induce papa to go to Paris and wait till you get fitted out there," said Alicia coldly.

"Well," Kitty spoke with reviving cheerfulness, "that is not a bad idea. But you won't like taking my place," she added shrewdly. "You will hate it even more than I."

"I never professed to like poverty and dirt and disease and immorality," said Alicia calmly. "I never said that they touched me or stirred my compassion, because they don't. Whatever I may be, I don't pretend."

"And I'm not so sure that Alicia doesn't pretend," said Kitty, when she had explained this change of front to Jack. Of course, like the lover he was, he was ready to go wherever his mistress commanded, and Homburg was jollier than the slums. "I think she's a greater humbug than I am," Kitty went on. "She professes to hate poor people, and to be going of mere curiosity—but you will see. She won't follow us to Homburg."

"I bet you anything she turns up in a week," said Jack confidently, but he was immediately snubbed.

When the holiday makers had departed, Alicia surveyed the position. It was August, and everybody was out of town—everybody who is anybody, that is to say; there are the nobodies who never have any holidays, but then they don't count. She debated within herself awhile how she should go to Baron Street, and she decided to drive. "I can keep the carriage and come back in it if I don't like the appearance of things," she argued as she dressed; and it was noticeable that she did not make a penitential toilet, as she had insisted that Kitty should do. She wore one of the Homburg costumes, and she put a flower at her throat.

"I am not one of them," she said with vehemence. "I will not stoop to make myself like them. If it were not that these people are our relatives, and that it would be extremely unpleasant if it came out that we let them die of starvation, I would not go near them."

On second thoughts she dismissed the carriage on reaching Baron Street. The door of the big house stood wide open, as it did at all hours of the night and day, and one or two idlers —men and women alike—were leaning against the walls and sitting on the doorsteps. The communistic principle is carried out very thoroughly in the East, and everything is done under the public eye. This may arise in part from the one-room system; when you share one apartment with your entire family, and perhaps a lodger or two thrown in, it becomes almost necessary to resort to the doorstep; and when you have nothing to do and nothing to eat, it is a natural instinct to fall back upon talk. The East-enders talk a great deal: it is their only amusement. The inhabitants of this particular street found a new topic in Alicia Frankland. They stared rather sullenly at the departing carriage and at the girl who remained—probably they took her for some more than usually gorgeous district visitor. She passed through among them and walked upstairs with a fearless step and a proud composure. He would have been a bold man who dared to be rude to her.

Up to the third floor she marched. The steps were worn and undeniably dirty. The open doors at various landings gave glimpses of interiors that might safely be called squalid. In one of these a bundle of dirty rags that presently proclaimed itself to be a woman lay prone across the threshold. She was no other, indeed, than the old lady who had frightened Kitty. From the opposite door came a fretting and wailing of children's voices. There are children enough, and they make noise enough—screaming, quarrelling, sobbing, sometimes, poor things, swearing; but how seldom does one hear them laugh!

Alicia passed on steadily till she came to a shut door on the third floor, and at that she knocked.

"Come in," said a pre-occupied voice, and turning the handle she entered. Mary sat at the window sewing fast, as if she were afraid the sunlight would absent itself at noon. For a moment Alicia had a chance of observing her unseen. The round, innocent, friendly country face was there still, but a new knowledge—a knowledge that was pain—had turned its sweetness sad. Mary looked up heavy-eyed as Alicia crossed the room with the step of a queen. Alicia did what she had probably never been known to do in her life before: she yielded to the promptings of a sudden kindly impulse. She stooped and kissed the pale, gentle face. She did not try to account to herself for the action afterwards. She chose to ignore it.

The tears, so long forbidden, came into Mary's eyes. There were no explanations—no, "I am Alicia," "and I Mary," because none were needed. It was impossible to be conventional after this. Alicia took the only remaining chair in the room, and lifted it to the opposite side of the table.

"I have come to spend part of the day with you if you will allow me," she said. "May I help you to sew?"

"It is rough work; it will hurt your hands. It is not for you."

Alicia contemplated her fingers with the sparkling rings on them. "Perhaps it will hurt them," she said; and she reached across, and lifted one of the coarse aprons from a pile Mary was hemming.

"Now," she said, "we can talk. But, first of all, I will tell you about myself—since papa has told us of you. I don't wish you to mistake me for a philanthropist. I am not at all a charitable person—I have no sympathy with district-visitors, or Sunday-school teachers, or sisters, or any of the people who give themselves up to good works. I don't pity the sorrows of the poor, or want to relieve them. I find my own troubles quite enough to manage, so I havn't come here to wash dirty children, or to talk to their mothers, or to preach the temperance gospel, or any other gospel. I have come because I am tired of my own class—I belong, I believe, to what are called the classes—and because I think it might amuse me to study the ways of the masses; that is why I am here, to amuse myself and pass the time."

Mary looked up with a little smile. That impulsive kiss had fatally flawed Alicia's argument.

"I can't tell you much," she said. "It would take a long time even to know a little, and we are strangers here. But I will tell you about the people in this house—those of them that I know. On the ground floor in the front room there is a young woman who supports a sickly, bedridden old father as a fitter of shirts. She gets her work direct from the shops, and being what is called a skilled worker, she can even earn enough at times to keep herself in comfort."

"She is not interesting then; prosperity is always prosaic; pass on."

"There are other sewing girls," said Mary, "who are not nearly so prosperous, I'm afraid. They go out and remain out all day, but they often look hungry and always tired. On the floor below this there is an old woman ——"

"I know," interrupted Alicia, "I saw her. She is a shameful old woman. I have no desire to know anything about her. She is one of the people who ought not to exist."

Mary looked at her a little sadly, but she said nothing. To her the wonder rather was that there were so few comparatively who consoled themselves for miseries untold after the fashion of this reprobate old person. "Opposite her," she continued, "is a young bricklayer with his wife and children—four of them in one little room. They came from the country six months ago. The children were rosy-cheeked then, I can well believe, and the mother tidy and neat. For the first three months or so the man had regular work, and even so much as twenty-four shillings a-week to spend; but for the last three months he has been unemployed. Four mouths to fill, and nothing to feed them with."

"It must have been his own fault," said Alicia hardly.

"No," said Mary, "it is because trade is dull, and there are more workers than work. You will hear the same story everywhere; you will grow very tired of hearing it."

"Why did he leave the country?"

"I suppose," said Mary slowly, "for the same reason that David and I did—because one thinks that everything can be accomplished in London. It is a cruel place," she said quietly and without bitterness, "cruel as death. It lures us here; it beguiles us with false dreams; and though we despair there is no going back—there is no going back."

"Is it not as easy to go back as it is to come?" said the young lady, who had never in her life known the check of an ungratified wish.

"In nine cases out of ten it isn't possible. The money is all spent for one thing; the furniture has been sold bit by bit for bread; the children's clothes have been pawned. Even if the money could be scraped together there is the shame to be faced—the shame of failure. The poor have their pride as well as the rich. Perhaps the going away has been against the advice of friends." Mary drooped over her work, and Alicia, looking at her, understood that it was her own experience on which she was inwardly dwelling. "When that has been the case, when everybody has been against your going, when all your friends have prophesied evil, it is harder to go back."

"That may be the case with the bricklayer, who ought certainly to have listened to the advice of his friends," said Alicia with severity, "but it can never be your position. When you are tired of living here"—she glanced round the bare room—"when you are tired of sewing aprons for—how much did you say?"

"For threepence a-dozen," said Mary, flushing under the question.

"For the handsome sum of threepence a-dozen! When, in short, you have had enough of this new play called London, you can go home once more. You will appreciate the country, I should think, doubly after this glimpse of a different life, and your experiment will give you something to think of when you happen to be dull."

Alicia spoke with a cutting coldness that would have been wounding if Mary's fine insight had not helped her to understand its motive. Alicia was saving Mary's pride and her own, by giving the Jardines journey to London the aspect of a masquerade—a caprice. "You and I are here to amuse ourselves, to pass the time," she seemed to say, with that proudly-held head of hers; "when we have had enough of it we shall go away and forget it."

Mary did not answer directly, but she said presently, "You don't know David."

"Ah," said Alicia, who had no pressing desire to know David; "you mean that he is growing tired of it already? I do not wonder at that. The streets must seem very irksome to one who has been used to the country, to fields, and sheep," she ended vaguely. "He is impatient, I suppose, after the manner of man, and wants to carry you off."

Mary shook her head.

"David will never go back," she said, sadly. "He has not repented coming here. I suppose it was too dull for him at home. When you see him you will understand. Nothing would make him go back; he will remain here till ——"

Till what? Till fame came to him with the laurel crown, or till death, easeful death, the ender of all strife, called him? Alicia scarcely cared to hear the answer. Her imagination remained cold over this poet-shepherd, this degenerate farmer, who had the perverted taste to prefer Baron Street to his own ploughed fields. Very likely while he dreamed himself a second Burns, he resembled that Scotch classic in nothing but his vices. Alicia did not care to pursue the subject of David; but, indeed, if Mary had meant to say more she could not have said it then, for the words were arrested on her trembling lips by a knock—bold, decided, almost imperious—at the door.

She started up, but Alicia was before her.

"I will go," she said. "You are tired. Sit still."

She moved across the room, and opened the door with a most majestic sweep. Miss Frankland could do nothing without being a little tragic.

"Miss Jardine," said a voice—a quiet and pleasant voice—"will you ——"

Here the speaker, who was a young man, became precipitately dumb, and fell back a step.

"Yes—it is I—Alicia Frankland," she said.

"But—to see you here!"

"Oh, pray don't think I have turned a charitable person," said Alicia. "That would be as strange a transformation as to rediscover Felix Mervyn in the guise of a philanthropist. You wish to see my cousin, Miss Jardine? She is here. Walk in."

(*To be continued.*)

